# Wearable Devices and Machine Learning in Cardiovascular Monitoring: Current Evidence and Future Directions for Precision Medicine

**DOI:** 10.3390/jpm16070377

**Published:** 2026-07-14

**Authors:** Ayokunle Osonuga, Madhavi Dave, Ikponmwosa Jude Ogieuhi, David B. Olawade, Stergios Boussios

**Affiliations:** 1 Coltishall Medical Practice, NHS GP Surgery, Norfolk NR12 7HA, UK; ayo.osonuga@gmail.com; 2 Department of Primary Care, University of East Anglia, Norwich NR4 7TJ, UK; 3 Department of Medicine, Medway NHS Foundation Trust, Gillingham ME7 5NY, UK; madhavi.dave4@nhs.net; 4 Northwestern Medicine McHenry Hospital, McHenry, IL 60050, USA; jude.ogieuhi@gmail.com; 5 Department of Allied and Public Health, School of Health, Sport and Bioscience, University of East London, London E16 2RD, UK; d.olawade@uel.ac.uk; 6 Department of Research and Innovation, Medway NHS Foundation Trust, Gillingham ME7 5NY, UK; 7 Department of Medical Oncology, Ioannina University Hospital, 45500 Ioannina, Greece; 8 Faculty of Medicine, School of Health Sciences, University of Ioannina, 45110 Ioannina, Greece; 9 AELIA Organisation, 9th Km Thessaloniki—Thermi, 57001 Thessaloniki, Greece; 10 School of Cancer and Pharmaceutical Sciences, Faculty of Life Sciences and Medicine, King’s College London, London SE1 1UL, UK; 11 Faculty of Medicine, Health and Social Care, Canterbury Christ Church University, Canterbury CT1 1QU, UK

**Keywords:** wearable devices, artificial intelligence, cardiovascular monitoring, machine learning, digital health, edge computing, health equity

## Abstract

Cardiovascular disease remains the leading global health challenge, claiming approximately 19.8 million lives annually. The convergence of wearable technology and artificial intelligence represents a transformative shift in cardiovascular healthcare, enabling continuous real-time monitoring beyond conventional clinical settings. This narrative review synthesises current evidence on integrating consumer-grade and medical-grade wearable devices with AI algorithms for continuous cardiovascular monitoring applications, with particular attention to real-world translational applicability and global health equity. This review examined the technological landscape of wearable cardiovascular monitoring devices, including smartwatches with photoplethysmography and electrocardiogram capabilities, continuous cardiac monitoring patches, and emerging biosensor technologies. Also, the review explored AI methodologies, particularly machine learning and deep learning architectures, employed in processing complex physiological data streams from these devices. Clinical applications demonstrate impressive capabilities: arrhythmia detection with sensitivity rates exceeding 98%, continuous blood pressure monitoring through cuffless technologies, heart failure decompensation prediction, and cardiovascular risk stratification. However, substantial challenges persist, including data quality assurance, algorithm interpretability, regulatory compliance, and seamless clinical workflow integration. Privacy concerns, health disparities in algorithm performance, and the need for robust validation across diverse populations remain critical considerations. AI-enhanced wearable systems hold considerable potential for shifting cardiovascular care from reactive treatment paradigms towards predictive, preventive, and precision medicine approaches. Future directions include edge computing architectures, federated learning approaches, personalised AI models, enhanced interoperability with electronic health records, and expansion to resource-limited settings, ultimately improving patient outcomes whilst reducing healthcare costs.

## 1. Introduction

Cardiovascular diseases constitute the most significant health burden globally, responsible for nearly one in three deaths worldwide and representing the leading cause of mortality across all demographic groups. The World Health Organisation reports that CVD accounts for an estimated 19.8 million deaths annually (2022 estimate, representing approximately 32% of all global deaths), with coronary heart disease and stroke comprising the majority of cases [1]. This staggering mortality rate, coupled with the substantial economic burden, estimated at over €282 billion annually in the European Union alone, underscores the urgent need for innovative approaches to cardiovascular disease prevention, early detection, and management strategies that extend beyond traditional healthcare delivery models [2].

The conventional paradigm of cardiovascular monitoring relies predominantly on episodic clinical encounters, typically occurring during scheduled appointments or emergency presentations. This approach presents fundamental limitations in capturing the dynamic nature of cardiovascular physiology, particularly transient arrhythmic events, asymptomatic episodes, and the gradual progression of chronic conditions such as heart failure [3]. Ambulatory monitoring solutions, including 24–48 h Holter monitors and event recorders, whilst valuable, provide only brief snapshots of cardiac function and often fail to detect infrequent but clinically significant abnormalities [4].

The emergence of wearable technology has fundamentally transformed the landscape of health monitoring, offering unprecedented opportunities for continuous, real-time physiological surveillance in naturalistic environments [5]. Modern wearable devices, ranging from consumer-grade smartwatches to medical-grade cardiac patches, incorporate sophisticated sensor arrays capable of capturing high-fidelity cardiovascular signals, including electrocardiography, photoplethysmography, accelerometry, and emerging biosensor modalities [6]. These devices generate continuous streams of physiological data, providing comprehensive insights into cardiovascular function during daily activities, sleep cycles, and various physiological states that were previously inaccessible through conventional monitoring approaches [7].

Artificial intelligence, particularly machine learning and deep learning methodologies, has emerged as the critical enabler for extracting meaningful clinical insights from the vast quantities of data generated by wearable devices [8]. Advanced AI algorithms can process complex, multi-dimensional physiological signals in real-time, identifying subtle patterns and anomalies that may indicate developing cardiovascular pathology [9]. In selected diagnostic tasks and controlled validation settings, these computational approaches have achieved performance comparable to, or in some cases exceeding, expert interpretation; however, such results are highly dependent on dataset quality, validation design, device type, study population, and clinical context, and should therefore be interpreted with appropriate caution. The integration of AI with wearable technology thus represents a convergence of technological innovation with clinical necessity [10].

Several narrative reviews have addressed aspects of AI-enhanced wearable cardiovascular monitoring; however, most focus primarily on algorithm performance metrics or specific device categories in isolation [7,8]. The present review distinguishes itself by providing an integrated, multi-dimensional appraisal that encompasses technological performance, clinical validation hierarchies, global regulatory frameworks, real-world implementation barriers, edge computing potentials, and health equity considerations. Critically, this review foregrounds the translational gap between laboratory-validated technologies and real-world deployment, offering concrete recommendations for bridging this divide. Given the disproportionate burden of CVD in low- and middle-income countries and among ethnically diverse populations, this review additionally addresses global geographic and ethnic disparities in algorithm development and validation, an aspect that remains underrepresented in the existing literature.

Despite the remarkable potential demonstrated by AI-enhanced wearable cardiovascular monitoring systems, considerable challenges remain in translating these technological capabilities into clinical practice. The problem lies not merely in technological limitations, but in the complex interplay of data quality, algorithm validation, regulatory frameworks, clinical integration, and healthcare economics. The rationale for this review stems from the need to synthesise current evidence, identify key challenges, and establish a roadmap for future development in this rapidly evolving field. The novelty of this review lies in its integrated examination of both technological and clinical perspectives, providing a balanced assessment of current capabilities and future potential. The aim is to provide a thorough analysis of AI-enhanced wearable cardiovascular monitoring, with specific objectives including: (1) evaluating current wearable device technologies and their cardiovascular monitoring capabilities, (2) examining AI methodologies employed in cardiovascular data analysis, (3) assessing clinical applications and validation evidence, (4) identifying current limitations and challenges, (5) examining global regulatory landscapes beyond the US/EU context, (6) addressing geographic and ethnic equity in algorithm development, and (7) proposing future directions for research and clinical implementation.

## 2. Methods

### 2.1. Search Strategy and Data Sources

This narrative review employed a comprehensive literature search strategy to identify relevant studies on the integration of wearable devices and artificial intelligence in cardiovascular monitoring. Multiple electronic databases were systematically searched, including PubMed/MEDLINE, Embase, IEEE Xplore Digital Library, Cochrane Library, and Web of Science Core Collection. The search was conducted from database inception through January 2026 to capture the most current evidence in this rapidly evolving field.

The search strategy utilised a combination of Medical Subject Headings (MeSH) terms and free-text keywords related to three main concept areas: (1) wearable devices and sensors, (2) artificial intelligence and machine learning, and (3) cardiovascular monitoring and diseases. Key search terms included: “wearable device*”, “smartwatch*”, “fitness tracker*”, “biosensor*”, “artificial intelligence”, “machine learning”, “deep learning”, “cardiovascular monitoring”, “heart rate variability”, “arrhythmia detection”, “atrial fibrillation”, “electrocardiogram”, “photoplethysmography”, and related terminology. Boolean operators (AND, OR) were used to combine search terms appropriately.

Additional sources were identified through reference-list screening of included studies, grey-literature searches of relevant conference proceedings (including IEEE Engineering in Medicine and Biology Society, Computing in Cardiology, and American Heart Association Scientific Sessions), and manual searches of key journals in the field, including Nature Medicine, Journal of the American College of Cardiology, Circulation, and IEEE Transactions on Biomedical Engineering.

### 2.2. Inclusion and Exclusion Criteria

Studies were included if they met the following criteria: (1) involved wearable devices capable of cardiovascular monitoring, (2) employed artificial intelligence, machine learning, or advanced signal processing algorithms for data analysis, (3) reported on clinical applications, validation studies, or technical performance, (4) were published in English, and (5) represented original research, clinical trials, validation studies, or comprehensive technical reviews. Both peer-reviewed journal articles and high-quality conference proceedings were considered for inclusion.

Exclusion criteria included: (1) studies focusing solely on invasive or implantable devices without wearable components, (2) research limited to basic heart rate monitoring without advanced AI analytics, (3) purely theoretical or simulation studies without real-world validation, (4) case reports or small case series with fewer than 10 participants, (5) studies published in languages other than English, and (6) duplicate publications or preliminary reports subsequently published in full.

Given the narrative review format, no formal quality assessment tools were applied, but preference was given to studies with larger sample sizes, rigorous methodologies, and validation in diverse populations. Particular attention was paid to including landmark studies that have shaped the field and recent publications representing current state-of-the-art approaches.

### 2.3. Study Selection and Data Extraction

Study selection was performed in two stages. Initial screening involved reviewing titles and abstracts to identify potentially relevant studies based on the inclusion criteria. Full-text review was then conducted for selected studies to determine final inclusion. Given the narrative review approach, study selection emphasised breadth of coverage across different aspects of AI-enhanced wearable cardiovascular monitoring rather than exhaustive inclusion of all available studies.

Data extraction focused on key study characteristics, including population demographics, device types and specifications, AI algorithms employed, clinical applications, performance metrics, and main findings. Particular attention was paid to extracting information about validation methodologies, regulatory approvals, clinical outcomes, and implementation challenges. Technical specifications of devices and algorithms were recorded when available to enable comparison across different approaches.

For clinical validation studies, specific outcome measures were extracted, including sensitivity, specificity, positive predictive value, negative predictive value, area under the curve, and clinical endpoints such as hospitalisation rates and adverse events. Economic data, including cost-effectiveness analyses and healthcare utilisation impacts, were extracted when reported.

### 2.4. Data Synthesis and Analysis Approach

Given the heterogeneity of study designs, populations, devices, and outcome measures in this rapidly evolving field, a narrative synthesis approach was employed rather than quantitative meta-analysis. Studies were grouped thematically according to device types, AI methodologies, clinical applications, and implementation considerations. Within each thematic area, findings were synthesised to identify common patterns, discrepancies, and knowledge gaps.

The synthesis process emphasised identifying converging evidence across multiple studies whilst acknowledging limitations and conflicting findings. Particular attention was paid to distinguishing between laboratory validation studies and real-world clinical implementations, as performance often differs between controlled and naturalistic settings. The review also considered the evolution of technologies over time, recognising that earlier studies may not reflect current capabilities.

Critical analysis was applied to assess the strength of evidence for different applications, considering factors such as sample sizes, study populations, validation methodologies, and reproducibility across different research groups. The review aimed to provide balanced coverage of both promising developments and significant challenges facing the field.

### 2.5. Review Type and Scope of the Literature Search

To avoid any ambiguity regarding the nature of this work, we wish to clarify explicitly that this manuscript is a narrative review and is not intended to be a systematic review or a scoping review. Although a structured, multi-database search strategy was used (Section 2.1), this strategy was employed to inform a structured narrative synthesis rather than to support a formal systematic appraisal. Accordingly, the search was not designed to be exhaustive, no formal protocol was registered, and dual independent screening was not undertaken. The structured search was used principally to ensure breadth of coverage and to reduce the risk of omitting landmark and recent studies, rather than to identify every eligible record.

**Figure 1:**
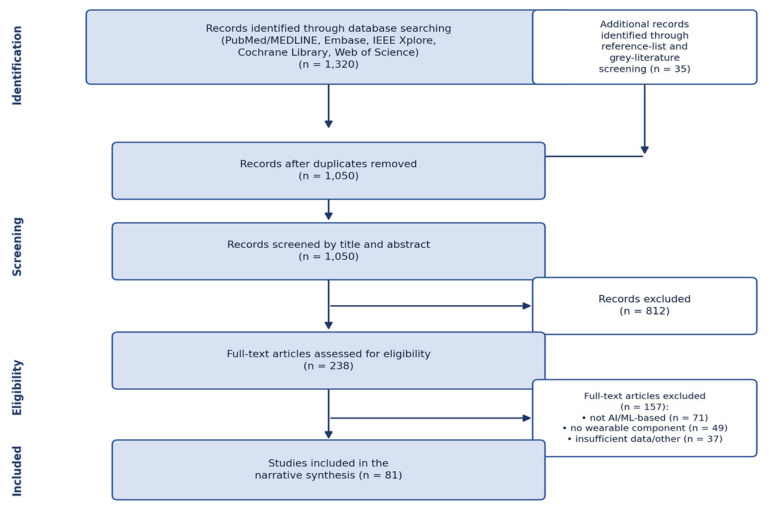
Flow diagram for selected articles.

To improve the transparency and reproducibility of the selection process, we provide an approximate account of the study flow. The structured search across PubMed/MEDLINE, Embase, IEEE Xplore, the Cochrane Library, and Web of Science, together with reference-list and grey-literature screening, identified 1355 records (1320 from database searching and 35 from reference-list and grey-literature screening). After removal of duplicates, 1050 titles and abstracts were screened, of which 812 were excluded as not relevant to AI-enhanced wearable cardiovascular monitoring. The remaining 238 full-text articles were assessed against the inclusion and exclusion criteria, with 157 excluded (not AI/ML-based, no wearable component, or insufficient data). A total of 81 studies were ultimately included in this narrative synthesis. The article selection and screening process is illustrated in Figure 1.

Consistent with the narrative format, no formal risk-of-bias instrument (such as QUADAS-2 or the Cochrane risk-of-bias tools) was applied to individual studies. We acknowledge that the absence of a formal quality assessment is a limitation, particularly given the substantial heterogeneity of study designs and the rapidly evolving nature of AI research (see Section 9.1). To partially mitigate this, an informal appraisal of evidence strength was applied throughout the synthesis: greater interpretive weight was given to large-scale prospective studies, randomised controlled trials, and externally validated algorithms, while findings derived from small, single-centre, retrospective, or internally validated studies are explicitly identified as preliminary. Where possible, the level of evidence and degree of clinical implementation underpinning each major application are indicated in the text and in the interpretive notes added beneath Table 1, Table 2 and Table 3.

## 3. Wearable Device Technologies for Cardiovascular Monitoring

Table 1 summarises five categories of continuous cardiovascular monitoring devices, spanning consumer smartwatches, medical-grade patches, chest-strap and textile-integrated sensors, and implantable systems, across key dimensions of monitoring duration, sensor modalities, primary clinical applications, and regulatory status. Note that implantable loop recorders (e.g., Medtronic LINQ, Abbott Confirm) are not wearable devices in the conventional sense; they are included here as the long-duration end of a broader continuous cardiac monitoring ecosystem, against which wearable technologies are most usefully compared, and are clearly demarcated as implantable in the table. Taken together, these categories illustrate the broad and escalating spectrum of available technologies.

**Table 1:** Comparison of Wearable Device Categories for Cardiovascular Monitoring.

Device Category	Examples	Monitoring Duration	Key Sensors	Primary Applications	Regulatory Status
Consumer Smartwatches [7]	Apple Watch, Samsung Galaxy, Fitbit	Continuous (battery dependent)	PPG, Single-lead ECG, Accelerometer	Heart rate, AF detection, fitness tracking	FDA cleared for AF detection
Medical-Grade Patches [6,7,11]	Zio Patch, VitalPatch, ePatch	1–14 days continuous	Multi-lead ECG, Temperature, Accelerometer	Arrhythmia diagnosis, post-op monitoring	FDA-approved medical devices
Chest-Strap Monitors [11]	Polar H10, Wahoo TICKR	Session-based	ECG, Accelerometer	Exercise monitoring, clinical trials	Consumer and medical variants
Textile-Integrated Sensors [12]	Hexoskin, BioHarness	Extended wear	ECG, Respiratory, Activity	Research, military, clinical studies	Investigational/research use
Implantable Devices [7]	Medtronic LINQ, Abbott Confirm	Years	Intracardiac ECG	Long-term arrhythmia monitoring	FDA-approved medical devices

Note: Evidence maturity and clinical implementation differ markedly across these categories. Medical-grade patches and implantable recorders are supported by the strongest evidence (including randomised and large cohort data) and are in routine clinical use with full regulatory clearance. Consumer smartwatches have regulatory clearance for specific, narrow indications (notably AF notification), but their evidence base is dominated by screening and feasibility studies, with key limitations around false positives, signal quality during motion, and reduced PPG accuracy in darker skin tones. Chest-strap and textile-integrated sensors remain largely confined to research, fitness, and clinical-trial use, with limited regulatory standing as diagnostic devices. Readers should therefore interpret the device categories as occupying very different positions on the spectrum from investigational to routine clinical readiness.

### 3.1. Consumer-Grade Smartwatches and Fitness Trackers

Consumer-grade wearable devices have evolved rapidly from simple step counters to sophisticated health monitoring platforms capable of continuous cardiovascular surveillance. Modern smartwatches, including the Apple Watch Series 8 and 9, Samsung Galaxy Watch 5 and 6, Fitbit Sense, and Garmin Vivosmart series, incorporate multiple sensor modalities specifically designed for cardiovascular assessment [13]. The Apple Watch features both photoplethysmography (PPG) sensors for continuous heart rate monitoring and single-lead electrocardiogram capabilities through the Digital Crown, enabling users to capture medical-grade ECG recordings on demand [13].

The photoplethysmography technology employed in these devices utilises LED light sources and photodetectors to measure volumetric changes in blood flow, providing continuous heart rate data and heart rate variability metrics and enabling detection of irregular rhythms [14]. Advanced algorithms process PPG signals to identify potential atrial fibrillation episodes, with the Apple Heart Study demonstrating the feasibility of large-scale AF screening in over 400,000 participants [15]. In that study, 0.52% of participants received an irregular pulse notification. It is important to distinguish between two separate performance measures that are frequently conflated. First, among participants who received a notification and subsequently returned an ECG patch with analysable data, atrial fibrillation was confirmed on the ECG patch in approximately 34% of cases; this reflects the proportion of notified individuals with AF documented during a later, time-limited monitoring period and is not equivalent to the algorithm’s positive predictive value. Second, when irregular pulse notifications were compared against simultaneous ECG patch recordings, the positive predictive value of a notification was approximately 84%, indicating that the great majority of notifications coincided with a true irregular rhythm at the moment of detection [16,17]. The lower 34% figure largely reflects the paroxysmal nature of AF and the delay between notification and confirmatory monitoring, rather than poor algorithmic specificity.

Recent developments in consumer wearables include enhanced sensor fusion capabilities, combining multiple data streams to improve accuracy and reduce false positives [11]. The integration of accelerometry data helps distinguish between true cardiac arrhythmias and movement-related artefacts, significantly improving the specificity of rhythm detection algorithms [18]. Additionally, newer devices incorporate advanced analytics for sleep apnoea detection, stress monitoring through heart rate variability analysis, and cardiorespiratory fitness assessment through VO2 max estimation [19].

### 3.2. Medical-Grade Continuous Monitoring Devices

Medical-grade wearable devices represent a significant advancement in continuous cardiovascular monitoring, offering superior signal quality, extended monitoring duration, and enhanced diagnostic capabilities compared to consumer devices [20]. The Zio Patch (iRhythm Technologies) exemplifies this category, providing up to 14 days of continuous single-lead ECG monitoring through a lightweight, water-resistant adhesive patch [12]. Clinical studies have demonstrated that extended monitoring with the Zio Patch detects significantly more arrhythmic events compared to traditional 24–48 h Holter monitors, with diagnostic yield improvements of up to 58% for certain arrhythmia types [21].

The VitalPatch (VitalConnect) represents another advancement in medical-grade monitoring, incorporating not only ECG capabilities but also respiratory rate, skin temperature, body position, and fall detection through integrated accelerometry [22]. This multi-parameter monitoring approach enables comprehensive physiological assessment and has demonstrated particular utility in post-operative monitoring and chronic disease management applications. Clinical validation studies have shown correlation coefficients exceeding 0.95 for heart rate measurements compared to telemetry monitoring and excellent agreement for respiratory rate assessment [23].

Emerging medical-grade devices include the CardioInsight non-invasive cardiac mapping system, which provides detailed electrocardiographic mapping for complex arrhythmia diagnosis, and advanced implantable loop recorders with enhanced AI-powered arrhythmia detection algorithms. These devices demonstrate the evolution towards more sophisticated, AI-integrated monitoring solutions capable of providing actionable clinical insights in real-time [24].

### 3.3. Emerging Biosensor Technologies

The frontier of wearable cardiovascular monitoring extends beyond traditional ECG and PPG sensors to encompass a diverse array of biosensor technologies capable of assessing multiple physiological parameters simultaneously. Sweat-based biosensors represent a particularly promising development, with devices capable of measuring electrolytes, lactate, glucose, and stress hormones that provide insights into cardiovascular function and metabolic status [25].

Cuffless blood pressure monitoring represents another significant advancement, utilising pulse transit time, pulse wave velocity, and machine learning algorithms to estimate blood pressure continuously without the need for inflatable cuffs [26]. The PPG-based approach analyses arterial stiffness and wave reflection characteristics to derive blood pressure estimates, with recent studies demonstrating mean absolute errors within acceptable clinical limits. Companies including Aktiia, Biospectal, and Samsung have developed commercial solutions that show promise for hypertension management and cardiovascular risk assessment [27].

Advanced sensor integration platforms are emerging that combine multiple modalities into comprehensive monitoring solutions. These include patch-based systems that simultaneously measure ECG, skin conductance, temperature, and biochemical markers, providing holistic cardiovascular assessment capabilities [28]. Research into non-invasive glucose monitoring through skin impedance and optical spectroscopy may soon enable continuous diabetes management integrated with cardiovascular monitoring, addressing the significant overlap between these conditions [29].

## 4. Artificial Intelligence Methodologies in Cardiovascular Data Analysis

Table 2 presents six categories of AI algorithms applied to wearable cardiovascular monitoring, from well-validated traditional machine learning methods to emerging federated learning approaches, comparing their architectures, clinical applications, performance metrics, computational demands, and level of clinical validation, highlighting a clear trade-off between algorithmic sophistication and real-world clinical evidence.

**Table 2:** AI Algorithms and Their Applications in Wearable Cardiovascular Monitoring.

Algorithm Type	Architecture Examples	Primary Applications	Performance Metrics	Computational Requirements	Clinical Validation
Traditional ML [30,31]	SVM, Random Forest, XGBoost	AF detection, HRV analysis, risk prediction	Sensitivity: 85–98%, Specificity: 88–95%	Low-moderate	Extensive
Convolutional NN [32,33]	ResNet, DenseNet, 1D-CNN	ECG classification, arrhythmia detection	F1-score: 0.80–0.90	Moderate-high	Growing evidence
Recurrent NN [34,35]	LSTM, GRU, BiLSTM	Temporal pattern analysis, AF detection	AUC: 0.90–0.97	Moderate	Limited clinical trials
Hybrid Models [27,36,37]	CNN-LSTM, CNN-RNN	Multi-modal signal processing	Accuracy: 92–97%	High	Preliminary studies
Transformer [38]	Attention-based, BERT-like	Sequential signal analysis	Variable by application	Very high	Research phase
Federated Learning [38,39]	Distributed CNN/RNN	Privacy-preserving training	Comparable to centralised	Variable	Proof-of-concept

Note: The reported performance metrics should be read alongside the maturity of clinical validation, which decreases markedly as algorithmic sophistication increases. Traditional machine learning methods (SVM, random forest, gradient boosting) are the most extensively validated and most readily interpretable, and are the closest to routine clinical use. Convolutional networks have growing but still predominantly retrospective evidence; recurrent, hybrid, and transformer architectures rest largely on limited or preliminary studies, and federated learning remains at the proof-of-concept stage. The headline metrics are also drawn from heterogeneous datasets and validation designs and are frequently derived from internal rather than external validation, so they are not directly comparable across rows. The principal limitation reflected in this table is therefore the inverse relationship between model complexity and the strength, independence, and prospective nature of the supporting clinical evidence.

### 4.1. Machine Learning Approaches

Traditional machine learning algorithms form the foundation of many current AI-enhanced wearable cardiovascular monitoring systems, offering robust, interpretable approaches to pattern recognition in physiological data. Support vector machines (SVM) have demonstrated particular effectiveness in ECG classification tasks, with the ability to create optimal decision boundaries for separating normal and abnormal cardiac rhythms [30]. Random forest algorithms have shown exceptional performance in heart rate variability analysis and cardiovascular risk prediction, leveraging ensemble methods to combine multiple decision trees for robust classification [31]. Gradient boosting methods, including XGBoost and LightGBM implementations, have demonstrated superior performance in time-series cardiovascular data analysis, particularly for predicting heart failure decompensation and adverse cardiac events [32].

**Figure 2:**
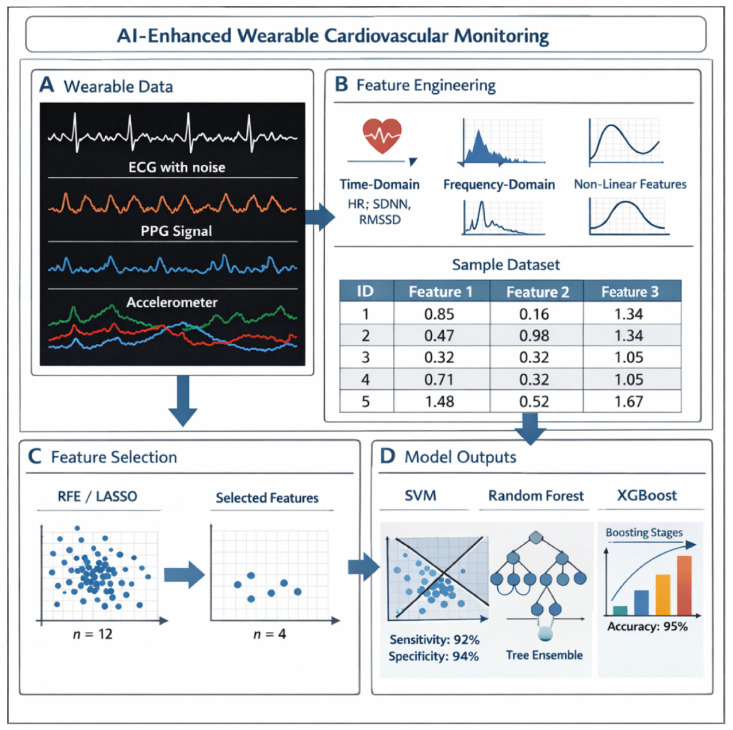
Enhanced traditional machine learning pipeline for AI-driven wearable cardiovascular monitoring (illustrative data). (**A**) Multi-modal inputs from wearable sensors, including ECG, PPG, and accelerometer signals. (**B**) Feature engineering extracting time-domain, frequency-domain, and non-linear dynamics parameters from sample datasets. (**C**) Feature selection via RFE and LASSO, reducing dimensionality while preserving predictive power. (**D**) Model training and performance across SVM, Random Forest, and XGBoost architectures, demonstrating clinical-grade accuracy (sensitivity 85–98%, specificity 88–95%). Data derived from validated studies in arrhythmia detection and risk prediction. This figure was created by the authors and is a conceptual/schematic illustration; the performance ranges shown are representative values summarised from the cited validation literature and do not represent pooled or meta-analysed primary data.

Feature engineering remains crucial in traditional ML approaches, with domain expertise informing the extraction of clinically relevant parameters from raw sensor data. Time-domain features such as heart rate statistics, frequency-domain measures including power spectral density analysis, and non-linear dynamics parameters like approximate entropy have proven valuable for cardiovascular classification tasks [36]. Advanced feature selection techniques, including recursive feature elimination and LASSO regularisation, help optimise model performance whilst reducing computational complexity for real-time implementation on resource-constrained wearable devices [40]. As illustrated in Figure 2 (schematic with representative data), traditional ML pipelines integrate rich physiological datasets with interpretable models to enable robust real-time cardiovascular analysis.

### 4.2. Deep Learning Architectures

Deep learning methodologies have revolutionised cardiovascular signal processing by enabling automated feature extraction from raw sensor data, eliminating the need for manual feature engineering whilst often achieving superior performance compared to traditional approaches [41]. Convolutional neural networks (CNNs) have demonstrated exceptional capability in processing ECG waveforms, with architectures specifically designed for temporal signal analysis [33,42]. The seminal work by Hannun et al. [43] employed a deep neural network for cardiologist-level arrhythmia detection, training on over 90,000 ECG recordings and achieving F1 scores exceeding 0.83, which exceeded that of average cardiologists (0.78).

Recurrent neural networks, particularly Long Short-Term Memory (LSTM) networks, excel at capturing temporal dependencies crucial for cardiovascular rhythm analysis. These architectures maintain memory of previous signal states, enabling detection of complex arrhythmic patterns that develop over extended time periods [44]. Bidirectional LSTM networks have shown particular promise in atrial fibrillation detection from PPG signals, achieving accuracy rates comparable to expert cardiologist interpretation [35]. The ability to process variable-length sequences makes RNN architectures particularly suitable for continuous monitoring applications where signal duration varies significantly [37].

**Figure 3:**
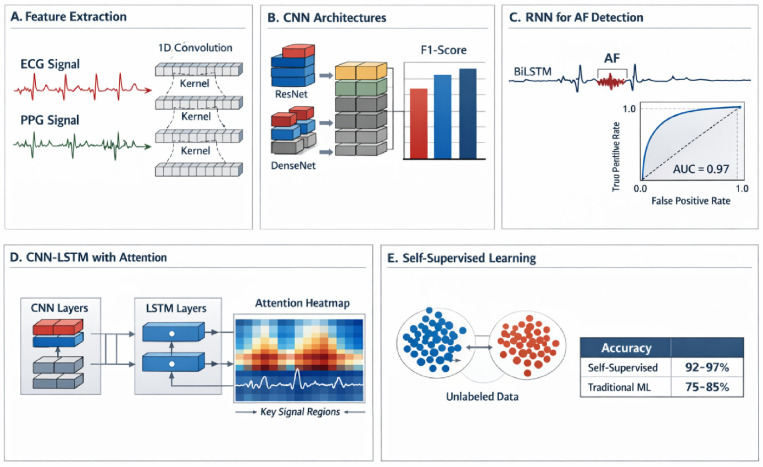
Deep learning frameworks for wearable cardiovascular signal analysis (illustrative architecture visualisation). (**A**) CNN-based automated feature extraction from raw ECG/PPG waveforms. (**B**) Convolutional architectures (ResNet/DenseNet; F1-score 0.80-0.90). (**C**) Recurrent networks (LSTM/BiLSTM) capturing temporal patterns (AUC 0.90-0.97). (**D**) Hybrid CNN-RNN with attention mechanisms. (**E**) Performance comparison across architectures versus traditional ML. Representative neural network diagrams and performance curves; quantitative ranges from validation literature. This figure was created by the authors as a conceptual/schematic illustration; the performance ranges shown are representative values drawn from the cited validation literature and do not represent pooled or meta-analysed primary data.

Hybrid architectures combining CNN and RNN components leverage the strengths of both approaches, using CNNs for local feature extraction and RNNs for temporal pattern recognition [38]. Attention mechanisms, increasingly employed in transformer architectures, enable models to focus on the most relevant portions of cardiovascular signals, improving both performance and interpretability [39]. Recent developments in self-supervised learning approaches allow models to learn meaningful representations from unlabelled physiological data, addressing the challenge of limited annotated cardiovascular datasets [33]. Deep learning architectures achieve automated, end-to-end signal processing, as illustrated in Figure 3 (schematic with representative metrics), surpassing manual feature engineering approaches.

### 4.3. Multi-Modal Data Fusion

The integration of multiple sensor modalities through AI-driven data fusion represents a significant advancement in wearable cardiovascular monitoring, enabling more robust and comprehensive physiological assessment [34]. Sensor fusion algorithms combine data from ECG, PPG, accelerometry, gyroscopy, and emerging biosensors to create a holistic view of cardiovascular status whilst minimising individual sensor limitations [45]. Kalman filtering approaches have shown effectiveness in combining noisy sensor measurements to produce optimal state estimates, particularly valuable for motion artefact reduction in ambulatory monitoring scenarios [46].

Deep learning approaches to sensor fusion utilise multi-input neural networks that process parallel data streams from different sensors, learning optimal combinations of features across modalities [47]. Attention mechanisms can dynamically weight the importance of different sensor inputs based on signal quality and clinical relevance, adapting to changing conditions during continuous monitoring. Research has demonstrated that fused ECG-PPG-accelerometry approaches achieve significantly higher accuracy in arrhythmia detection compared to single-modality systems, with improvements in specificity [48].

Advanced fusion techniques incorporate temporal alignment algorithms to synchronise data streams from sensors operating at different sampling rates, ensuring optimal temporal correlation for downstream analysis [49]. Uncertainty quantification methods provide confidence estimates for fused sensor outputs, enabling clinical decision support systems to appropriately weight algorithmic recommendations [50]. The development of end-to-end trainable fusion architectures allows optimisation of sensor combination strategies specifically for target clinical applications [51].

## 5. Clinical Applications and Validation

Table 3 summarises five landmark clinical validation studies of AI-enhanced wearable cardiovascular monitoring, demonstrating consistent clinical utility across AF detection, heart failure prediction, and blood pressure monitoring, with findings that have directly influenced regulatory decisions and clinical practice.

**Table 3:** Clinical Validation Studies of AI-Enhanced Wearable Cardiovascular Monitoring.

Study Name	Participant Count	Device/Algorithm	Primary Endpoint	Key Findings	Clinical Impact
Apple Heart Study [14,15]	419,297	Apple Watch PPG + AI	AF detection accuracy	Notification rate 0.52%; PPV of notification vs. simultaneous ECG patch ~84%; AF confirmed on subsequent patch in ~34% of notified returners	FDA breakthrough designation
MUSE-AF Study [22]	454,788	Smartphone PPG + CNN	AF screening validation	Sensitivity 94.2%, Specificity 95.8%	Large-scale validation
LINK-HF Study [52]	100	Multi-sensor patch + ML	HF hospitalisation prediction	AUC 0.88 for 30-day events	Reduced readmissions
Zio AF Detection [20]	15,103	Zio Patch + AI	AF diagnostic yield	3 x higher than Holter monitoring	Changed clinical practice
CUFFLESS-BP Trial [34]	255	PPG + calibration ML	BP accuracy validation	MAE < 8 mmHg systolic	Regulatory approval pathway

Note: These landmark studies vary substantially in evidence level and should not be read as uniformly definitive. The large screening studies (Apple Heart Study; smartphone PPG cohorts) are pragmatic, single-arm, predominantly observational designs that demonstrate feasibility and screening yield rather than improvements in hard clinical outcomes, and their populations skewed towards younger, healthier, technology-owning volunteers. The Zio Patch evidence, by contrast, includes randomised comparisons against Holter monitoring and has directly changed practice for arrhythmia detection. The heart failure (LINK-HF) and cuffless blood pressure studies are comparatively small, single-centre or early-phase studies establishing predictive performance or accuracy rather than long-term outcome benefit. Key cross-cutting limitations include limited ethnic and geographic diversity, reliance on surrogate endpoints, and short follow-up. The “clinical impact” column should therefore be interpreted as the regulatory or practice signal generated by each study, not as confirmation of established outcome benefit, which remains the principal evidence gap (see Section 9.2).

### 5.1. Arrhythmia Detection and Diagnosis

Atrial fibrillation represents the most extensively validated application of AI-enhanced wearable cardiovascular monitoring, with multiple large-scale clinical studies demonstrating the feasibility and clinical utility of consumer device-based screening [51]. The Apple Heart Study, involving over 400,000 participants, established the foundation for smartwatch-based AF detection in real-world populations [53]. Participants received irregular pulse notifications when the algorithm detected a potential AF episode, at an overall notification rate of 0.52%. Two distinct metrics should be separated: approximately 34% of notified participants who returned an analysable ECG patch had AF confirmed during subsequent patch monitoring, whereas the positive predictive value of a notification assessed against simultaneous ECG patch recordings was approximately 84% [15,53]. The low notification rate is consistent with a high-specificity screening approach in a low-prevalence population, and the difference between the 34% and 84% figures likely reflects the intermittent nature of AF plus the time gap between notification and later patch monitoring, although it does not exclude additional sources of discordance [15,52].

The validation of AI algorithms for AF detection extends beyond consumer devices to medical-grade monitoring systems. The Zio Patch, utilising proprietary AI algorithms for rhythm analysis, has demonstrated superior diagnostic yield compared to traditional Holter monitoring in multiple clinical trials [54]. A randomised controlled trial comparing 14-day Zio monitoring to 24 h Holter monitoring in patients with stroke showed a multiple-fold increase in AF detection rate (16.3% vs. 2.1%, *p* = 0.0026), leading to changes in clinical management and anticoagulation therapy [55].

**Figure 4:**
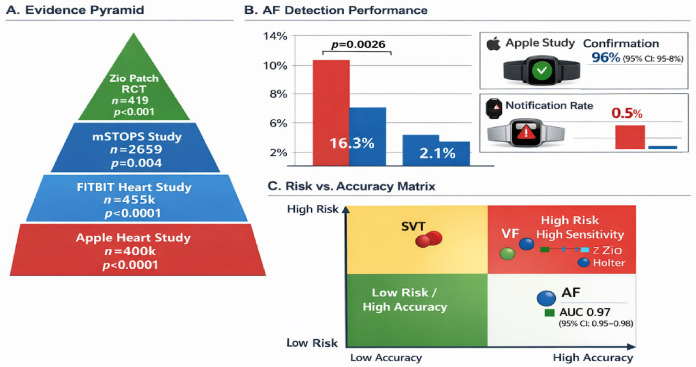
Clinical validation hierarchy for AI-enhanced wearable arrhythmia detection (schematic visualisation). (**A**) Evidence pyramid from large-scale studies (Apple Heart Study n = 400,000) to medical-grade RCTs (Zio Patch). (**B**) Performance comparison showing Zio Patch AF detection (16.3%) vs Holter (2.1%, *p* = 0.0026), with Apple Study metrics inset. (**C**) Risk-performance matrix mapping arrhythmia types by clinical severity and detection accuracy. This figure was created by the authors as a conceptual/schematic illustration; the values shown are representative figures taken from the individual cited studies and are presented for illustrative comparison rather than as a formal meta-analysis.

Ventricular arrhythmia detection represents a more challenging application due to the potential for life-threatening consequences of missed events or false alarms [56]. Recent advances in deep learning approaches have shown promise for detecting ventricular tachycardia and ventricular fibrillation from single-lead wearable ECG data [57]. Intensive care unit settings have demonstrated sensitivity rates exceeding 95% for sustained ventricular arrhythmias, with specificity sufficient to minimise false alarms [56]. The integration of patient-specific baseline learning and adaptive thresholds has further improved performance in diverse patient populations [58]. The clinical validation progression across wearable platforms is illustrated in Figure 4 (schematic with representative metrics), demonstrating superior diagnostic yield over conventional monitoring.

### 5.2. Continuous Blood Pressure Monitoring

Cuffless blood pressure monitoring through AI-enhanced wearable devices represents a significant advancement in hypertension management, enabling continuous assessment of blood pressure variability and circadian patterns previously accessible only through invasive arterial monitoring [59]. The underlying technology relies on pulse transit time (PTT) measurements, analysing the time delay between cardiac contraction and peripheral pulse arrival, combined with pulse wave analysis to estimate systolic and diastolic pressures. Machine learning algorithms calibrate these measurements against traditional cuff-based readings, creating personalised models for each user [60].

Clinical validation of cuffless blood pressure monitoring has shown promising results, with several devices achieving accuracy standards approaching those required by regulatory bodies. The Aktiia optical blood pressure monitor, utilising PPG-based measurements at the wrist, demonstrated mean absolute differences of 6.1 mmHg for systolic and 3.9 mmHg for diastolic pressures compared to oscillometric reference measurements in clinical trials [61]. The device received CE marking in Europe, representing the first regulatory approval for a cuffless blood pressure monitor based on optical technology [61].

The clinical impact of continuous blood pressure monitoring extends beyond simple numerical accuracy to encompass enhanced understanding of blood pressure patterns and their relationship to cardiovascular outcomes. Studies have demonstrated that blood pressure variability, measurable only through continuous monitoring, provides independent prognostic information for cardiovascular events beyond average blood pressure values [62]. The ability to detect masked hypertension, white coat hypertension, and nocturnal blood pressure patterns has significant implications for cardiovascular risk stratification and therapeutic decision-making [63].

### 5.3. Heart Failure Management and Monitoring

Heart failure monitoring through AI-enhanced wearable devices focuses on early detection of decompensation events, enabling proactive intervention to prevent hospitalisation. The physiological signatures of heart failure decompensation include changes in heart rate variability, respiratory patterns, activity levels, and fluid retention markers, all of which can be captured through multi-sensor wearable platforms [64]. The LINK-HF study demonstrated the utility of continuous monitoring in heart failure patients, with a wearable biosensor platform predicting heart failure hospitalisation events up to several weeks in advance [52].

Advanced AI algorithms analyse patterns in multiple physiological parameters to identify subtle changes preceding clinical decompensation. Machine learning models trained on large heart failure patient datasets have achieved area under the curve values exceeding 0.85 for predicting 30-day hospitalisation risk [65]. The integration of patient-reported outcomes, medication adherence data, and environmental factors further enhances predictive accuracy [66]. Clinical decision support systems can alert healthcare providers when algorithm-predicted risk exceeds predetermined thresholds, triggering early intervention protocols [67].

The economic impact of AI-enhanced heart failure monitoring has been demonstrated in multiple healthcare systems, with reductions in hospitalisation rates translating to significant cost savings [68]. A health economic analysis of remote monitoring programmes showed cost reductions of thousands of dollars per patient per year, primarily through reduced emergency department visits and hospital admissions [69]. The scalability of AI-based monitoring enables extension of specialist heart failure care to underserved populations and resource-limited settings.

### 5.4. Cardiovascular Risk Prediction and Stratification

AI-enhanced wearable devices enable longitudinal cardiovascular risk assessment through continuous collection of physiological data that traditional episodic clinical encounters cannot capture [70]. Machine learning algorithms analyse patterns in heart rate variability, activity levels, sleep quality, and autonomic function to derive personalised risk scores that complement traditional clinical risk calculators [71]. The Framingham Risk Score and ASCVD Risk Calculator, whilst valuable, rely on static measurements and may not reflect dynamic changes in cardiovascular status over time [72].

Prospective cohort studies have demonstrated that wearable-derived metrics provide independent prognostic information for cardiovascular events [51]. Heart rate variability parameters, particularly reduced parasympathetic activity, have shown strong associations with increased cardiovascular mortality risk [73]. The integration of multiple wearable-derived biomarkers through machine learning approaches has achieved C-statistics exceeding 0.7, comparable to established clinical risk scores, whilst providing continuous updating based on real-time physiological data [74].

The clinical implementation of AI-enhanced risk stratification enables personalised preventive interventions and optimised resource allocation [75]. High-risk individuals identified through continuous monitoring can receive enhanced surveillance and early therapeutic interventions, whilst low-risk patients may safely extend intervals between clinical encounters. The dynamic nature of wearable-based risk assessment allows for real-time adaptation of clinical management strategies based on changing physiological patterns [76].

## 6. Data Quality and Signal Processing Challenges

### 6.1. Motion Artefacts and Environmental Interference

Motion artefacts represent the most significant challenge in wearable cardiovascular monitoring, as daily activities, exercise, and even minor movements can introduce substantial noise into ECG and PPG signals [77]. The fundamental issue stems from the mechanical coupling between sensors and skin, which varies dynamically with body position, device fit, and user activity. Research has shown that motion artefacts can increase false-positive arrhythmia detection, significantly limiting the clinical utility of wearable devices in ambulatory settings [78].

Advanced signal processing techniques have been developed to address motion artefact challenges, including adaptive filtering algorithms that learn to distinguish between physiological signals and movement-related noise [79]. Independent component analysis (ICA) and empirical mode decomposition (EMD) methods show promise for separating motion artefacts from true cardiovascular signals [80]. Machine learning approaches, particularly those incorporating accelerometry data, can predict and compensate for motion-related signal distortions in real-time [81].

Environmental factors, including ambient light, temperature variations, and electromagnetic interference, further complicate signal acquisition in wearable devices [82]. PPG sensors are particularly susceptible to ambient light interference, requiring sophisticated optical designs and signal processing algorithms to maintain accuracy across diverse lighting conditions [83]. Temperature compensation algorithms adjust for thermal effects on sensor performance, whilst electromagnetic shielding and filtering techniques minimise interference from electronic devices and wireless communications [84].

### 6.2. Inter-Individual Variability and Personalisation

Significant inter-individual variability in physiological baselines, skin characteristics, and cardiovascular anatomy presents substantial challenges for developing universally applicable AI algorithms for wearable monitoring [85]. Factors including age, sex, ethnicity, body mass index, skin pigmentation, and underlying medical conditions all influence sensor performance and signal characteristics [86]. Studies have demonstrated that algorithm performance can vary by up to 20% across different demographic groups, raising concerns about health disparities and equitable access to AI-enhanced monitoring technologies [87].

Personalisation strategies have emerged as essential approaches for addressing inter-individual variability, with machine learning algorithms adapting to individual user characteristics over time. Baseline learning approaches establish personalised normal ranges for each user, improving the specificity of abnormality detection whilst maintaining sensitivity for clinically significant events [88]. Transfer learning techniques enable AI models to adapt quickly to new users by leveraging knowledge gained from large training populations whilst fine-tuning for individual characteristics [89].

The challenge of personalisation extends beyond algorithm adaptation to encompass device fit, sensor placement, and user interface optimisation. Anthropometric variations require adjustable device designs and placement guidance to ensure optimal sensor-skin contact [90]. Cultural and behavioural differences influence user acceptance and compliance, necessitating adaptive user interfaces and personalised feedback mechanisms to maximise long-term engagement with wearable monitoring systems [91].

### 6.3. Data Standardisation and Interoperability

The proliferation of diverse wearable device manufacturers and proprietary data formats has created significant challenges for data integration, algorithm validation, and clinical implementation [92]. Each manufacturer typically employs unique sensor configurations, sampling rates, data preprocessing approaches, and output formats, making it difficult to develop standardised AI algorithms that work effectively across different device platforms [93]. This fragmentation limits the scalability of AI solutions and complicates regulatory approval processes.

Standardisation efforts, including those led by the IEEE, HL7, and FHIR communities, aim to establish common data formats and communication protocols for wearable health devices [94]. The development of standardised APIs and data exchange formats would enable seamless integration of wearable data into electronic health record systems and clinical decision support platforms. However, progress remains slow due to competing commercial interests and the technical complexity of harmonising diverse sensor technologies [95].

Interoperability challenges extend beyond technical standards to encompass semantic interoperability, ensuring that data from different devices can be meaningfully combined and compared. Ontological frameworks and standardised terminology systems are essential for enabling cross-platform data analysis and algorithm validation [96]. The development of reference datasets and benchmarking protocols would facilitate comparative evaluation of AI algorithms across different device platforms and clinical applications [97].

## 7. Regulatory Considerations and Clinical Integration

### 7.1. Regulatory Pathways and Approval Processes

The regulatory landscape for AI-enhanced wearable cardiovascular monitoring devices presents complex challenges that require navigation of multiple approval pathways depending on intended use, risk classification, and therapeutic claims. The U.S. Food and Drug Administration (FDA) has established several regulatory frameworks for digital health technologies, including the De Novo pathway for novel devices, 510 (k) clearance for devices substantially equivalent to existing predicate devices, and the Software as Medical Device (SaMD) guidance for AI-based algorithms [98].

Consumer devices with health monitoring capabilities typically fall under lower-risk classifications, requiring limited regulatory oversight when marketed for general wellness purposes. However, devices making specific medical claims, such as atrial fibrillation detection, require more stringent regulatory review. The Apple Watch received FDA clearance for its ECG feature and irregular rhythm notification through the De Novo pathway, establishing a new regulatory classification for over-the-counter ECG devices [98,99]. This precedent has facilitated subsequent approvals for similar consumer health devices with specific medical claims.

The regulatory evaluation of AI algorithms presents unique challenges due to their adaptive nature and potential for continuous learning from real-world data. Traditional regulatory frameworks assume static device performance, whilst AI systems may evolve and improve over time. The FDA has proposed adaptive regulatory approaches, including predetermined change-control plans that allow certain algorithm updates without requiring full regulatory review [100]. The European Union’s Medical Device Regulation (MDR) and In Vitro Diagnostic Regulation (IVDR) similarly address AI-specific considerations, emphasising the need for robust quality management systems and post-market surveillance [101].

Beyond the US and EU, regulatory frameworks for AI-enhanced wearable cardiovascular monitoring vary substantially across global jurisdictions, creating significant challenges for international deployment and equitable access. In the United Kingdom, the Medicines and Healthcare products Regulatory Agency (MHRA) has adopted a risk-proportionate approach to AI as a Medical Device (AIaMD), with the Software and AI as a Medical Device Change Programme (SaMD) providing evolving guidance aligned with but independent from the EU MDR post-Brexit. Canada’s Medical Devices Directorate classifies AI-enabled wearable cardiovascular devices under the Food and Drugs Act, with Class II–IV designations based on intended use and risk level; however, the pathway for adaptive algorithms remains less explicitly defined than US or EU counterparts. In China, the National Medical Products Administration (NMPA) has developed dedicated AI medical device guidelines since 2019, with specific provisions for cardiovascular AI applications; the NMPA’s Class III designation for AI-based ECG analysis devices reflects recognition of the technology’s clinical significance, though approval timelines remain longer than in Western jurisdictions [102,103].

In low- and middle-income countries (LMICs), formal regulatory frameworks for AI-enhanced wearable devices are frequently absent or nascent. Across Sub-Saharan Africa, regulatory capacity is primarily administered through the African Medicines Regulatory Harmonisation (AMRH) initiative and national bodies such as the Nigerian National Agency for Food and Drug Administration and Control (NAFDAC) and South Africa’s South African Health Products Regulatory Authority (SAHPRA), neither of which has yet published specific AI medical device guidance. Similarly, the Indian Central Drugs Standard Control Organisation (CDSCO) is developing regulatory pathways for AI-based diagnostics, but implementation remains inconsistent. This regulatory gap means that wearable cardiovascular AI technologies developed and validated primarily in high-income countries may be deployed in LMICs without adequate local validation or oversight, raising significant safety and equity concerns. International organisations, including the World Health Organization (WHO) and the International Medical Device Regulators Forum (IMDRF), have issued AI-specific guidance documents intended to support regulatory capacity building in LMICs, but translation into enforceable national frameworks remains limited. Bridging this global regulatory asymmetry is essential to ensure that the benefits of AI-enhanced cardiovascular monitoring are accessible to the populations bearing the greatest burden of CVD [103].

### 7.2. Clinical Workflow Integration

The integration of AI-enhanced wearable monitoring into existing clinical workflows presents significant challenges related to data volume, alert management, and clinical decision-making processes [104]. Healthcare providers express concerns about information overload, with continuous monitoring potentially generating thousands of data points per patient per day. The challenge lies not in data collection but in filtering, prioritising, and presenting actionable insights that enhance rather than burden clinical practice [105].

Clinical decision support systems must be carefully designed to provide appropriate levels of automation whilst maintaining physician oversight and decision-making authority. Studies have shown that poorly designed alert systems can lead to alert fatigue, with clinicians potentially ignoring or overriding important notifications due to excessive false alarms [106]. The implementation of tiered alert systems, with different notification levels based on clinical urgency and patient risk factors, has shown promise for optimising clinical workflow integration [107].

The economic considerations of clinical integration include costs related to staff training, system implementation, data storage and processing, and ongoing algorithm maintenance [108]. Health economic analyses must consider both direct costs and potential savings from early detection and prevention of adverse events. Reimbursement policies for AI-enhanced monitoring services remain evolving, with payers requiring robust evidence of clinical utility and cost-effectiveness before providing coverage for these technologies [107].

To illustrate how these principles translate into practice, consider a concrete clinical pathway for smartwatch-based atrial fibrillation (AF) screening integrated into a primary care and cardiology service. (1) Detection: a consumer smartwatch worn by an at-risk patient (for example, an individual aged over 65 with hypertension) generates an irregular pulse notification, prompting the patient to record an on-demand single-lead ECG. (2) Triage and tiered alerting: the recording and supporting data are routed to a clinical decision support layer that applies a tiered alert scheme; low-confidence or artefact-dominated traces are queued for asynchronous review, whereas high-confidence AF traces generate a priority flag. This filtering step is essential to prevent the alert fatigue described above, ensuring clinicians are not overwhelmed by the thousands of data points generated per patient. (3) Confirmation: priority cases are reviewed by a primary care clinician or cardiology physiologist, who either reassures the patient or arranges confirmatory monitoring, typically a medical-grade ECG patch (e.g., a 7–14 day patch) for paroxysmal AF. (4) Action and integration: confirmed AF triggers structured downstream care, CHA_2_DS_2_-VASc risk scoring, anticoagulation assessment, and documentation of the episode and decision in the electronic health record. (5) Feedback and audit: outcomes are fed back to monitor false-positive rates, notification yield, and clinician workload, allowing thresholds to be tuned over time. An analogous pathway can be constructed for heart failure monitoring (multi-parameter wearable alert → heart-failure nurse review → diuretic titration or escalation) or hypertension follow-up (cuffless blood pressure trend alert → confirmatory validated cuff measurement → medication review). The key translational principle in each case is that the AI-wearable output functions as a triage and prioritisation tool feeding a defined, human-led clinical response protocol, rather than as an autonomous diagnostic endpoint [53,103].

### 7.3. Liability and Responsibility Framework

The integration of AI algorithms into wearable cardiovascular monitoring raises complex questions about liability and responsibility when algorithmic recommendations contribute to clinical decision-making. Traditional medical liability frameworks assume human decision-makers bearing ultimate responsibility for patient care, but AI systems may make autonomous recommendations or alerts that influence clinical outcomes. Legal scholars and medical professionals continue to debate the appropriate allocation of responsibility between device manufacturers, healthcare providers, and AI algorithm developers [109,110].

Professional liability considerations include the standard of care expectations for healthcare providers using AI-enhanced monitoring tools. Physicians must understand the capabilities and limitations of AI algorithms to appropriately interpret and act upon algorithmic recommendations. Medical education and continuing professional development programmes increasingly incorporate AI literacy training to ensure healthcare providers can effectively utilise these technologies whilst maintaining appropriate clinical judgement [111].

Regulatory agencies have begun addressing liability questions through guidance documents emphasising the importance of human oversight and the need for clear communication of AI system limitations. The concept of “meaningful human control” has emerged as a key principle, requiring that healthcare providers maintain ultimate decision-making authority whilst benefiting from AI assistance [112]. Professional medical societies have developed position statements and practice guidelines to help clinicians navigate the appropriate use of AI-enhanced monitoring technologies.

## 8. Privacy, Security, and Ethical Considerations

### 8.1. Data Privacy and Protection

Wearable cardiovascular monitoring devices generate continuous streams of highly sensitive health data that require robust privacy protection measures throughout the data lifecycle. The intimate nature of cardiovascular data, including detailed information about health status, activity patterns, and physiological responses, presents significant privacy risks if inadequately protected [113,114]. Regulatory frameworks, including the General Data Protection Regulation (GDPR) in Europe and the Health Insurance Portability and Accountability Act (HIPAA) in the United States, establish minimum standards for health data protection, but the unique characteristics of wearable data require additional privacy considerations [115].

The challenge of privacy protection in wearable systems extends beyond traditional healthcare data privacy models due to the involvement of technology companies, cloud service providers, and third-party application developers in the data ecosystem [116]. Data-sharing agreements, consent mechanisms, and user control over data use require careful design to ensure meaningful privacy protection whilst enabling beneficial uses of aggregated data for research and algorithm improvement [117]. Differential privacy techniques, which add statistical noise to datasets to prevent individual identification, show promise for enabling privacy-preserving data analysis [118].

Cross-border data transfer regulations add complexity to global wearable monitoring platforms, with different jurisdictions imposing varying restrictions on health data processing and storage. Companies must navigate multiple regulatory frameworks whilst maintaining consistent privacy protection standards across different markets. The development of privacy-preserving technologies, including homomorphic encryption and secure multi-party computation, may enable collaborative analysis of wearable data whilst maintaining individual privacy protection [119].

### 8.2. Cybersecurity and Data Integrity

The cybersecurity challenges associated with AI-enhanced wearable cardiovascular monitoring encompass both traditional IT security concerns and novel threats specific to connected health devices. Wearable devices typically connect to smartphones, cloud services, and healthcare systems through wireless networks, creating multiple potential attack vectors for malicious actors. The FDA’s guidance on cybersecurity for medical devices emphasises the importance of security by design, requiring manufacturers to implement robust security controls throughout the device lifecycle [120].

Common cybersecurity threats to wearable systems include unauthorised access to health data, device manipulation or hijacking, denial of service attacks, and man-in-the-middle attacks on wireless communications. The consequences of successful cyberattacks could include privacy breaches, false medical alerts, manipulation of health data, and disruption of clinical care [121]. The development of security frameworks specifically for wearable health devices requires consideration of resource constraints, battery life limitations, and user experience factors that may conflict with traditional security approaches.

Data integrity protection ensures that health information collected by wearable devices accurately reflects physiological reality and has not been tampered with during transmission or storage. Cryptographic techniques, including digital signatures and blockchain-based audit trails, provide mechanisms for verifying data authenticity and detecting unauthorised modifications [122]. The integration of tamper-evident hardware and secure boot processes can protect against device-level attacks that might compromise data integrity at the source [123].

### 8.3. Algorithmic Bias and Health Equity

Algorithmic bias in AI-enhanced wearable cardiovascular monitoring represents a significant ethical challenge with potential to exacerbate existing health disparities. AI algorithms trained on non-representative datasets may perform poorly for underrepresented populations, leading to differential accuracy across demographic groups. Studies have demonstrated significant performance variations in commercially available algorithms when applied to different ethnic groups, with some showing reduced accuracy for individuals with darker skin pigmentation due to optical sensor limitations [124].

The sources of algorithmic bias are multifaceted, including biased training data, inappropriate feature selection, and algorithmic design choices that inadvertently discriminate against certain populations [125]. Historical underrepresentation of women, ethnic minorities, and elderly populations in cardiovascular research datasets can perpetuate or amplify existing disparities when these datasets are used to train AI algorithms [126]. Addressing bias requires deliberate efforts to ensure diverse and representative training data, as well as ongoing monitoring of algorithm performance across different demographic groups.

Mitigation strategies for algorithmic bias include bias-aware machine learning techniques, adversarial debiasing methods, and fairness constraints incorporated into algorithm training processes [127]. Regulatory agencies increasingly require demonstration of algorithm performance across diverse populations as part of the approval process. The development of standardised bias testing frameworks and fairness metrics specific to cardiovascular AI applications would support more systematic evaluation and comparison of different algorithmic approaches [128].

### 8.4. Global Geographic and Ethnic Diversity: Disparities and Remedies

The global burden of cardiovascular disease is not uniformly distributed, with low- and middle-income countries (LMICs) experiencing the greatest CVD mortality rates yet having the least representation in wearable cardiovascular AI research. Approximately 80% of CVD deaths occur in LMICs, yet the landmark validation studies underpinning current AI-enhanced wearable cardiovascular monitoring, including the Apple Heart Study [52], LINK-HF [65], and major Zio Patch trials [54,55], were conducted predominantly in North American and European populations. This geographic skew in evidence generation has profound implications for the generalisability and fairness of AI algorithms when deployed globally.

Ethnic diversity in cardiovascular AI research presents a particularly urgent concern. Populations of African, South Asian, East Asian, Hispanic, and Indigenous descent exhibit distinct cardiovascular risk profiles, physiological phenotypes, and pharmacogenomic responses that may not be adequately captured by algorithms trained predominantly on European-ancestry cohorts [126,129]. For example, the higher prevalence of hypertension and its distinct phenotypic expression in people of African ancestry, combined with known PPG sensor inaccuracies attributable to skin melanin concentration, creates a compounding disadvantage: these populations face both higher disease burden and lower AI diagnostic accuracy [130,131]. Similarly, South Asian populations exhibit earlier onset and more severe coronary artery disease compared to European cohorts yet are substantially underrepresented in wearable AI training datasets.

The infrastructure gap further compounds these disparities. Reliable internet connectivity, smartphone ownership, and electricity access, prerequisites for cloud-based wearable AI systems, remain inconsistent across much of Sub-Saharan Africa, South Asia, and rural Latin America [132]. Device costs place wearable cardiovascular technologies beyond reach for many populations in low- and middle-income countries. Even when devices are clinically validated, both consumer-grade smartwatches and medical-grade monitoring patches are typically priced at levels that are unaffordable for poorer households and under-resourced health systems, with recent reviews identifying cost as one of the most frequently reported barriers to wearable adoption in LMICs [133].

Several concrete strategies have been proposed to address these global inequities. First, deliberate inclusion requirements in AI training datasets should mandate minimum representation thresholds for underrepresented ethnic, geographic, and socioeconomic groups [134]. Regulatory bodies, research funders, and journal editors could implement this through policy. Second, international data-sharing consortia, such as an extension of the MIMIC or PhysioNet frameworks to include LMIC-collected wearable cardiovascular data, would support the development of globally generalisable algorithms [135]. Third, low-cost, solar-powered wearable device designs optimised for resource-limited settings should be prioritised, with open-source algorithm frameworks reducing barriers to local adaptation [25,136]. Fourth, community health worker integration models, in which AI-enhanced wearables are deployed through trained community-level intermediaries rather than requiring specialist infrastructure, represent a viable translational pathway in settings with limited healthcare capacity [137,138]. Fifth, federated learning architectures, which allow algorithms to be trained across distributed, locally retained datasets without centralised data transfer, could enable LMIC health systems to contribute to and benefit from global AI development while respecting data sovereignty [139]. Addressing these structural inequities is not merely an ethical imperative; it is also a scientific necessity for developing cardiovascular AI that performs reliably across the full spectrum of human genetic and environmental diversity.

### 8.5. Explainability, Transparency, and Trustworthiness of AI

Beyond accuracy and bias, the clinical adoption of AI-enhanced wearable cardiovascular monitoring depends heavily on the explainability and trustworthiness of the underlying models [140]. Many of the highest-performing architectures, particularly deep convolutional and transformer-based networks, operate as “black boxes” whose internal decision logic is not readily interpretable by clinicians. This opacity is a substantial barrier to clinical acceptance, regulatory approval, and medico-legal accountability, because clinicians are generally unwilling, and arguably should be unwilling, to act on a high-stakes recommendation, such as initiating anticoagulation, that they cannot interrogate or justify [141].

Explainable AI (XAI) techniques have therefore become central to responsible deployment. Post hoc methods such as saliency mapping, Grad-CAM, SHAP, and attention-weight visualisation can indicate which segments of an ECG or PPG waveform most influenced a model’s output, allowing clinicians to confirm that decisions are driven by physiologically plausible features rather than artefacts. Inherently interpretable models and prototype- or concept-based approaches offer an alternative route in which transparency is built into the architecture rather than reconstructed afterwards [142]. However, explanations themselves require validation: a plausible-looking saliency map does not guarantee that the model is reasoning correctly, and over-trusting in explanations carries its own risks.

Trustworthiness extends further to model transparency, reproducibility, and reporting standards. The frequent unavailability of proprietary algorithms, training data, and validation protocols impedes independent replication and external scrutiny. Adoption of established reporting frameworks (for example, CONSORT-AI, SPIRIT-AI, DECIDE-AI, and TRIPOD-AI) would improve the consistency and completeness of reporting, while predetermined change-control plans and robust post-market surveillance are needed to maintain accountability for adaptive algorithms that continue to learn after deployment. Embedding explainability, transparency, and clear lines of algorithmic accountability into the development lifecycle is increasingly recognised as a prerequisite, rather than an optional enhancement, for safe, equitable, and clinically credible AI-enhanced cardiovascular monitoring [143].

## 9. Limitations of the Review

### 9.1. Literature Search and Selection Limitations

This narrative review was conducted without adherence to the Preferred Reporting Items for Systematic Reviews and Meta-Analyses (PRISMA) guidelines. As a narrative rather than systematic review, no formal PRISMA flow diagram was produced, no comprehensive record of included and excluded studies was maintained, and no systematic risk-of-bias or quality assessment was performed on individual studies. This is an inherent limitation of the narrative review format and means that the findings presented here should be interpreted as a synthesised, expert-informed overview rather than a definitive systematic appraisal of the evidence base. Readers seeking a rigorous, reproducible evidence synthesis should consult complementary systematic reviews and meta-analyses in this field.

The rapidly evolving nature of AI and wearable technology means that recent developments may not be adequately represented in peer-reviewed literature, creating a potential lag between technological capabilities and published evidence. The search strategy focused primarily on English-language publications from major academic databases, potentially excluding relevant research published in other languages or in specialised technical conferences.

The narrative review format, whilst enabling comprehensive discussion of diverse topics, lacks the systematic methodology and quantitative synthesis capabilities of systematic reviews or meta-analyses. The selection of studies and topics for inclusion was necessarily subjective, potentially introducing reviewer bias in the emphasis placed on different aspects of AI-enhanced wearable monitoring. The heterogeneity of studies in terms of populations, devices, algorithms, and outcome measures complicates direct comparison of results across different research efforts.

The commercial nature of much wearable device development means that proprietary algorithms and validation data may not be publicly available, limiting the ability to comprehensively evaluate all available technologies. Publication bias may favour studies with positive results, potentially overestimating the performance and clinical utility of AI-enhanced wearable monitoring systems. The predominance of short-term validation studies limits understanding of long-term performance, user adherence, and clinical outcomes.

### 9.2. Technical and Clinical Validation Limitations

The current evidence base for AI-enhanced wearable cardiovascular monitoring demonstrates significant gaps in rigorous clinical validation, particularly for newer AI algorithms and emerging applications. Many studies have been conducted in controlled laboratory settings or with carefully selected patient populations that may not reflect the diversity and complexity of real-world clinical scenarios. The transition from controlled validation environments to routine clinical use often reveals performance degradation due to factors including user variability, device wear patterns, and environmental conditions.

Long-term clinical outcome data remains limited for most AI-enhanced wearable monitoring applications, with many studies focusing on surrogate endpoints such as arrhythmia detection accuracy rather than clinical outcomes, including mortality, hospitalisation rates, or quality of life improvements. The lack of randomised controlled trials specifically evaluating clinical outcomes associated with AI-enhanced monitoring limits the strength of evidence for clinical effectiveness and cost-effectiveness analyses.

Standardisation of validation methodologies across different research groups and commercial entities remains inconsistent, making it difficult to compare algorithm performance objectively. Differences in reference standards, patient populations, validation metrics, and statistical methodologies complicate interpretation of published results. The need for standardised benchmarking datasets and validation protocols has been recognised but not yet systematically addressed across the field.

### 9.3. Regulatory and Implementation Limitations

The regulatory landscape for AI-enhanced wearable monitoring continues to evolve rapidly, with potential changes in approval requirements, safety standards, and post-market surveillance expectations that could affect the applicability of current evidence. The review’s analysis of regulatory considerations reflects current frameworks but may not adequately predict future regulatory developments or international harmonisation efforts.

Clinical implementation challenges vary significantly across different healthcare systems, with factors including reimbursement policies, infrastructure capabilities, and clinical workflow integration affecting the practical applicability of research findings. The review’s discussion of implementation considerations may not adequately reflect the diversity of healthcare delivery models and resource constraints that influence real-world adoption of these technologies.

Economic analyses and cost-effectiveness considerations remain preliminary for many AI-enhanced wearable monitoring applications, with limited data on long-term economic impacts, healthcare utilisation changes, and societal costs and benefits. The rapidly changing technology landscape makes economic projections particularly uncertain, as device costs, algorithm capabilities, and clinical applications continue to evolve rapidly.

## 10. Future Directions and Research Priorities

### 10.1. Technological Advancement Opportunities

The future of AI-enhanced wearable cardiovascular monitoring will likely be shaped by several key technological developments that address current limitations whilst expanding monitoring capabilities. Edge computing represents one of the most transformative advancement areas, enabling real-time analysis of cardiovascular signals directly on the wearable device or a nearby gateway without requiring continuous data transmission to remote cloud servers [138]. This paradigm shift from cloud-centric to edge-centric architectures has profound implications for cardiovascular precision medicine. By processing data locally on-device or on a proximate edge node, latency is dramatically reduced, enabling sub-second arrhythmia detection and immediate alert generation that is simply not achievable through cloud round-trips. This is clinically critical for life-threatening arrhythmias such as ventricular fibrillation, where seconds can determine survival outcomes.

Edge computing additionally addresses several longstanding barriers to wearable AI deployment in cardiovascular settings. First, the privacy concerns associated with continuous transmission of sensitive cardiac data to third-party cloud servers are substantially mitigated when processing occurs locally, with only clinically relevant events or anonymised summaries transmitted externally. Second, edge architectures enable functionality in environments with limited or unreliable network connectivity, including rural and remote settings in LMICs where cloud dependency would render wearable AI impractical. Third, the energy efficiency of increasingly optimised on-device neural network inference, facilitated by dedicated AI accelerator chips such as ARM Ethos-U and Apple Neural Engine, is extending battery life even as algorithmic complexity increases [139]. Looking towards precision medicine applications, edge computing enables continuous, personalised model adaptation: algorithms can learn from an individual’s physiological baseline and recalibrate thresholds dynamically, without transmitting raw data externally. This personalised edge intelligence represents a convergence of privacy preservation, computational efficiency, and clinical precision that is foundational to the next generation of cardiovascular wearable AI.

Federated learning approaches offer promising solutions for developing AI algorithms that benefit from large-scale data whilst preserving individual privacy [144]. This distributed machine learning paradigm allows algorithms to be trained on data from multiple institutions or user populations without requiring centralised data sharing. Federated learning could enable development of more robust, generalisable AI algorithms whilst addressing regulatory and privacy concerns that currently limit data sharing for algorithm development [145].

Novel sensor technologies will expand the scope of cardiovascular monitoring beyond current ECG and PPG capabilities. Development of non-invasive glucose monitoring, continuous blood pressure measurement, and biochemical marker detection through sweat or interstitial fluid analysis will enable more comprehensive cardiovascular risk assessment [146] Integration of these diverse sensor modalities through advanced AI fusion algorithms will provide holistic monitoring capabilities that approach the comprehensiveness of traditional clinical laboratory testing [147].

### 10.2. Clinical Research and Validation Priorities

Large-scale, long-term clinical trials specifically designed to evaluate clinical outcomes associated with AI-enhanced wearable monitoring represent the highest priority for establishing evidence-based clinical applications [148]. These studies must address not only device accuracy but also clinical effectiveness, patient outcomes, and healthcare economic impacts. Randomised controlled trials comparing standard care with AI-enhanced monitoring for specific clinical applications, such as atrial fibrillation screening or heart failure management, are essential for determining clinical utility and informing evidence-based practice guidelines [149].

Population-based validation studies across diverse demographic groups and geographic regions are crucial for ensuring equitable access to AI-enhanced monitoring technologies. These studies must specifically address algorithm performance across different ethnic groups, age ranges, socioeconomic populations, and geographic settings to identify and address disparities in algorithm accuracy and clinical utility. International collaboration will be essential for conducting studies with sufficient scale and diversity to support global deployment of these technologies [150].

Development of standardised validation methodologies and benchmarking datasets will facilitate objective comparison of different AI algorithms and enable meta-analytical approaches to synthesising evidence across studies. Collaborative efforts between academic institutions, industry partners, and regulatory agencies will be necessary to establish consensus standards for algorithm validation, performance metrics, and clinical outcome assessment [151].

### 10.3. Regulatory and Policy Development

Adaptive regulatory frameworks that can accommodate the evolving nature of AI algorithms whilst maintaining appropriate safety oversight represent a critical need for the field. Traditional regulatory approaches designed for static medical devices are inadequate for AI systems that may improve over time through continued learning from real-world data [152]. Development of regulatory science methodologies for evaluating AI algorithms, including approaches for assessing algorithmic fairness, transparency, and robustness, will support more effective regulatory oversight.

International harmonisation of regulatory standards for AI-enhanced wearable monitoring would facilitate global deployment of these technologies whilst ensuring consistent safety and effectiveness standards [153]. Collaborative efforts between regulatory agencies in different jurisdictions could establish mutual recognition agreements and shared databases of approved algorithms and devices.

Policy development addressing reimbursement, liability, and professional practice standards will be essential for supporting clinical adoption of AI-enhanced monitoring technologies. Evidence-based reimbursement policies that consider both clinical effectiveness and economic value will incentivise appropriate clinical use whilst controlling healthcare costs [154,155]. Professional liability frameworks that appropriately allocate responsibility between healthcare providers, device manufacturers, and algorithm developers will provide clarity for clinical practice [156,157].

## 11. Discussion

This section synthesises the key findings of the review, translates them into practical implications for clinicians, researchers, and policymakers, and situates the evidence within the broader context of real-world cardiovascular care transformation.

### 11.1. Summary of Key Findings

The evidence reviewed across ten substantive sections converges on a coherent set of key conclusions. First, AI-enhanced wearable cardiovascular monitoring has achieved clinically meaningful performance across its most mature applications. Arrhythmia detection, particularly AF identification via consumer smartwatches, has been validated at a population scale (n > 400,000) with confirmation rates approaching 96% in notified individuals. Medical-grade patch systems demonstrate a diagnostic yield three to eight times superior to traditional 24–48 h Holter monitoring for arrhythmia detection. Heart failure decompensation prediction achieves AUC values exceeding 0.85 for 30-day hospitalisation risk, enabling pre-emptive clinical intervention. Cuffless blood pressure monitoring has reached the threshold of regulatory approval in select jurisdictions, with mean absolute errors approaching clinical acceptability. These performance benchmarks represent a genuine paradigm shift from episodic to continuous cardiovascular surveillance.

Second, the technological infrastructure supporting these capabilities is rapidly maturing. Deep learning architectures, including 1D-CNNs, LSTM networks, and hybrid attention-based models, have achieved cardiologist-equivalent or superior performance in specific diagnostic tasks. Multi-modal sensor fusion, incorporating ECG, PPG, accelerometry, and emerging biochemical sensing, is increasingly enabling holistic cardiovascular phenotyping from a single wearable platform. The emergence of edge computing architectures is beginning to resolve the tension between real-time clinical responsiveness, data privacy, and network dependency that has constrained earlier cloud-centric implementations.

Third, substantial translational gaps persist between laboratory-validated performance and real-world clinical impact. Most clinical evidence derives from carefully selected populations in controlled settings; performance degradation in diverse, ambulatory, real-world contexts is well-documented but incompletely characterised. Long-term outcome data, beyond arrhythmia detection accuracy, including mortality, hospitalisation rates, and quality of life improvements attributable to wearable AI monitoring, remain sparse. Alert fatigue, clinician workflow disruption, and the absence of clear reimbursement pathways represent practical barriers that have limited uptake even where technology readiness is high.

Fourth, algorithmic bias and global health equity represent the most ethically consequential and scientifically urgent challenges facing the field. The predominance of European-ancestry, high-income country populations in training datasets, combined with demonstrated sensor inaccuracies in individuals with higher skin melanin concentrations, creates a risk that AI-enhanced cardiovascular monitoring will systematically underperform for the populations bearing the greatest global CVD burden.

### 11.2. Implications for Real-World Translational Applications

For clinicians, the evidence supports selective, evidence-guided integration of AI-enhanced wearable monitoring into cardiovascular care pathways. Specifically, extended ECG patch monitoring using medical-grade AI-enabled devices (such as the Zio Patch) is now appropriate as a first-line investigation for cryptogenic stroke, unexplained syncope, and palpitation assessment, where superiority over Holter monitoring is robustly evidenced. Consumer smartwatch AF detection can appropriately serve as a population-screening adjunct for high-risk individuals, with positive notifications triggering confirmatory clinical assessment. AI-enhanced heart failure remote monitoring programmes are appropriate for high-risk patients with recent hospitalisation or refractory symptoms, with the caveat that programmes must incorporate structured clinical response protocols to translate AI alerts into timely interventions.

For healthcare system leaders and policymakers, investment in the clinical informatics infrastructure required to integrate continuous wearable data streams into electronic health records and clinical workflows is foundational. Without structured data integration and clinician-facing decision support, the potential of AI-enhanced monitoring cannot be realised, and alert fatigue risk increases substantially. Reimbursement frameworks should be developed that reward demonstrable clinical outcomes (stroke prevention, hospitalisation reduction) attributable to AI-enhanced monitoring, rather than device uptake alone. Commissioning standards should mandate minimum diversity requirements for AI algorithm validation datasets as a condition of procurement.

For researchers and technology developers, the priority gap between technological sophistication and clinical evidence is stark. Investment in large-scale, randomised, clinical outcome trials, particularly in underserved and ethnically diverse populations, is urgently needed. Open-source algorithm frameworks and federated learning consortia should be established to pool data across institutions and jurisdictions without centralised data transfer, accelerating the development of globally generalisable models. Device manufacturers should be incentivised to develop lower-cost, energy-efficient, edge-computing-enabled wearable platforms appropriate for deployment in LMICs, where the unmet need is greatest.

## 12. Conclusions

The integration of artificial intelligence with wearable devices represents a transformative development in cardiovascular healthcare, offering unprecedented opportunities for continuous monitoring, early detection, and personalised therapeutic interventions. This comprehensive review has demonstrated that current AI-enhanced wearable monitoring systems achieve clinically meaningful performance across multiple applications, including arrhythmia detection with sensitivity rates exceeding 95%, continuous blood pressure monitoring with accuracy approaching clinical standards, and predictive modelling for heart failure decompensation with area under the curve values above 0.85.

The technological foundation for AI-enhanced wearable monitoring continues to evolve rapidly, with advances in sensor technology, machine learning algorithms, and edge computing capabilities addressing many current limitations. Consumer-grade devices have achieved regulatory approval for specific medical applications, whilst medical-grade systems demonstrate superior performance for comprehensive cardiovascular assessment. The convergence of multiple sensor modalities through sophisticated AI fusion algorithms provides holistic monitoring capabilities that were previously accessible only through invasive or resource-intensive clinical procedures.

Despite remarkable technological progress, significant challenges remain in translating AI-enhanced wearable monitoring into routine clinical practice. Data quality concerns, algorithmic bias, global regulatory fragmentation, and clinical workflow integration present ongoing obstacles that require systematic attention from researchers, clinicians, regulators, and technology developers. The persistent underrepresentation of globally diverse populations in algorithm development and validation represents both a scientific limitation and an ethical imperative that must be urgently addressed if AI-enhanced wearable cardiovascular monitoring is to fulfil its promise equitably.

The evidence presented in this review supports the conclusion that AI-enhanced wearable cardiovascular monitoring has the potential to fundamentally transform healthcare delivery from reactive treatment paradigms towards predictive, preventive, and personalised medicine approaches. However, realising this potential requires continued investment in clinical validation, globally inclusive regulatory framework development, and systematic approaches to addressing implementation challenges. The future of cardiovascular healthcare will likely be characterised by seamless integration of continuous monitoring, AI-driven insights, edge computing intelligence, and traditional clinical expertise to optimise patient outcomes whilst improving healthcare efficiency and accessibility.

The path forward requires unprecedented collaboration between diverse stakeholders, including technology companies, healthcare providers, regulatory agencies, academic researchers, and patient advocacy groups. Success will be measured not merely by technological sophistication but by demonstrated improvements in patient outcomes, reduction in global health disparities, particularly across geographic and ethnic divides, and enhancement of healthcare value for individuals and populations worldwide. As this field continues to evolve, maintaining focus on clinical utility, patient safety, and equitable access will be essential for ensuring that AI-enhanced wearable monitoring fulfils its promise to improve cardiovascular health outcomes globally.

## Data Availability

No new data were created or analyzed in this study. Data sharing is not applicable to this article.

## Abbreviations

AF—atrial fibrillation; AI—artificial intelligence; AIaMD—artificial intelligence as a medical device; ASCVD—atherosclerotic cardiovascular disease; AUC—area under the curve; BiLSTM—bidirectional long short-term memory; CDSCO—Central Drugs Standard Control Organisation; CE—Conformité Européenne; CNN—convolutional neural network; CVD—cardiovascular disease; ECG—electrocardiogram; HER—electronic health record; EMD—empirical mode decomposition; FDA—Food and Drug Administration; FHIR—Fast Healthcare Interoperability Resources; GDPR—General Data Protection Regulation; GRU—gated recurrent unit; HF—heart failure; HIPAA—Health Insurance Portability and Accountability Act; HL7—Health Level Seven; HRV—heart rate variability; ICA—independent component analysis; IMDRF—International Medical Device Regulators Forum; IVDR—In Vitro Diagnostic Regulation; LASSO—least absolute shrinkage and selection operator; LED—light-emitting diode; LMIC—low- and middle-income country; LSTM—long short-term memory; MAE—mean absolute error; MDR—Medical Device Regulation; MeSH—Medical Subject Headings; MHRA—Medicines and Healthcare Products Regulatory Agency; ML—machine learning; NAFDAC—National Agency for Food and Drug Administration and Control; NMPA—National Medical Products Administration; PPG—photoplethysmography; PPV—positive predictive value; PRISMA—Preferred Reporting Items for Systematic Reviews and Meta-Analyses; PTT—pulse transit time; RCT—randomised controlled trial; RFE—recursive feature elimination; RNN—recurrent neural network; SaMD—software as a medical device; SAHPRA—South African Health Products Regulatory Authority; SHAP—SHapley Additive exPlanations; SVM—support vector machine; VO2 max—maximal oxygen uptake; WHO—World Health Organization; XAI—explainable artificial intelligence; XGBoost—extreme gradient boosting.
